# The “Wet Collar” Sign: A Case of Paraphimosis on CT

**DOI:** 10.7759/cureus.24345

**Published:** 2022-04-21

**Authors:** Jordan R Davis, Gina T Baaklini, Ryan B Schwope

**Affiliations:** 1 Department of Radiology, Brooke Army Medical Center, San Antonio, USA; 2 Department of Urology, Brooke Army Medical Center, San Antonio, USA

**Keywords:** “wet collar”, radiology, imaging, ct, urologic emergency, paraphimosis

## Abstract

Paraphimosis is a urologic emergency that requires prompt diagnosis to avoid potential morbidity. Diagnosis is made clinically and imaging findings have not been described. We will present the case of an 84-year-old man with a history of metastatic prostate cancer and prior pelvic external beam radiation therapy who presented to the emergency department with urinary retention. A urethral Foley catheter was placed for bladder decompression. He subsequently developed painful penile swelling and was found to have iatrogenic paraphimosis. Retrospective review of his contrast-enhanced CT of the abdomen and pelvis performed while in the emergency department before hospital admission revealed the relatively thickened, hypoattenuating prepuce located proximal to the corona of the glans penis, consistent with the clinical diagnosis. We will examine the imaging findings in this case and propose the novel “wet collar” sign to suggest the diagnosis of paraphimosis on CT.

## Introduction

Paraphimosis is a urologic emergency and prompt detection and treatment must occur in order to avoid morbidity, which can ultimately include necrosis of penile tissues and the necessity for operative management [1-2]. It is diagnosed by physical examination and imaging has not had a role in evaluation. As a result, imaging protocols have not been developed nor have imaging findings been described in the literature. Herein we present the case of an 84-year-old man who presented to our institution with urinary retention who developed paraphimosis after placement of a urinary catheter. A CT which was performed for other reasons was later retrospectively found to have signs which correlated with his clinical diagnosis. We will describe the imaging findings in our case and submit a novel imaging sign of paraphimosis for consideration that could merit further study.

## Case presentation

An 84-year-old man with a complicated medical history to include metastatic prostate cancer previously treated with pelvic external beam radiation therapy whose course was complicated by a right ureteral stricture treated with an internal ureteral stent presented to our emergency department with 14 hours of urinary retention. He was tachycardic but afebrile. A Foley catheter was placed by the nursing staff 18 hours after symptom onset which yielded one liter of urine. Concurrent blood analysis indicated leukopenia and neutropenia. Urinalysis demonstrated pyuria. Blood and urine cultures were obtained. Given his clinical presentation, an antibiotic regimen was initiated for presumptive bacteremia with a urinary source, and a contrast-enhanced CT of the abdomen and pelvis was obtained to assess for any evidence of other complications, including hydronephrosis.

CT showed a mildly hydronephrotic right kidney with a delayed nephrogram and asymmetric cortical atrophy, as well as an appropriately positioned internal right ureteral stent and the presumed site of stricture within the mid ureter; however, the acuity of these findings could not be determined as comparison images were not available. The urinary bladder was adequately decompressed. There were otherwise no immediately suspicious genitourinary findings, and eventually the patient was admitted to the internal medicine service to continue antibiotic treatment while awaiting results from the blood and urine cultures.

The following afternoon, almost 24 hours after Foley catheter placement, he was noted to have penile swelling and complained of sensitivity to touch, stating that his hospital gown and sheets were causing him discomfort when they rubbed against his genitals. At that time he was documented to have iatrogenic paraphimosis for which the primary team initially planned to prescribe a course of non-steroidal anti-inflammatory medicine to decrease penile swelling and allow the prepuce to reduce. However, early the next morning, nearly 35 hours after Foley catheter placement, urology was consulted due to progressive penile swelling and pain, and his paraphimosis was manually reduced. He was later discharged from the hospital after his blood and urine cultures were negative for greater than 48 hours. His episode of acute urinary retention and pyuria was likely multifactorial but upon further investigation was found to be a recurring problem for which he sought treatment with his primary medical facility.

**Figure 1:**
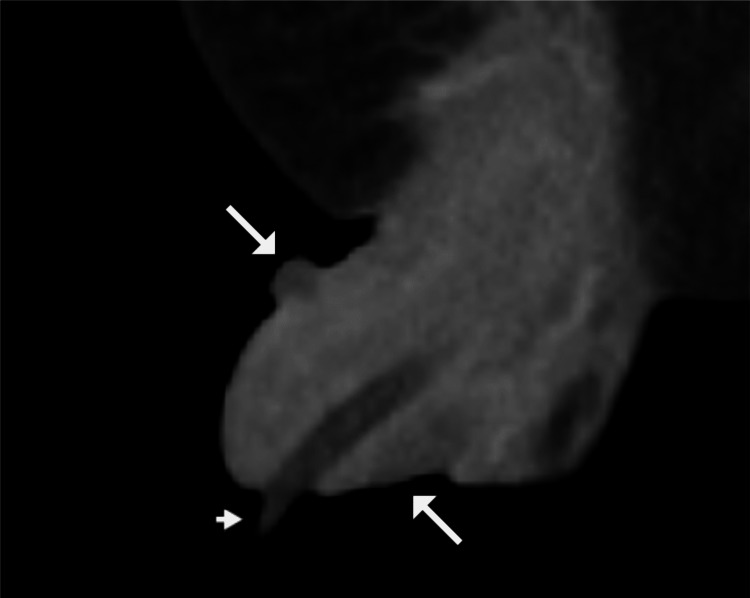
Coronal contrast-enhanced CT of the penis. Coronal contrast-enhanced CT of the penis shows a mildly thickened and hypoattenuating prepuce located proximal to the glans penis (long arrows) and a Foley catheter in the urethra (short arrow).

**Figure 2:**
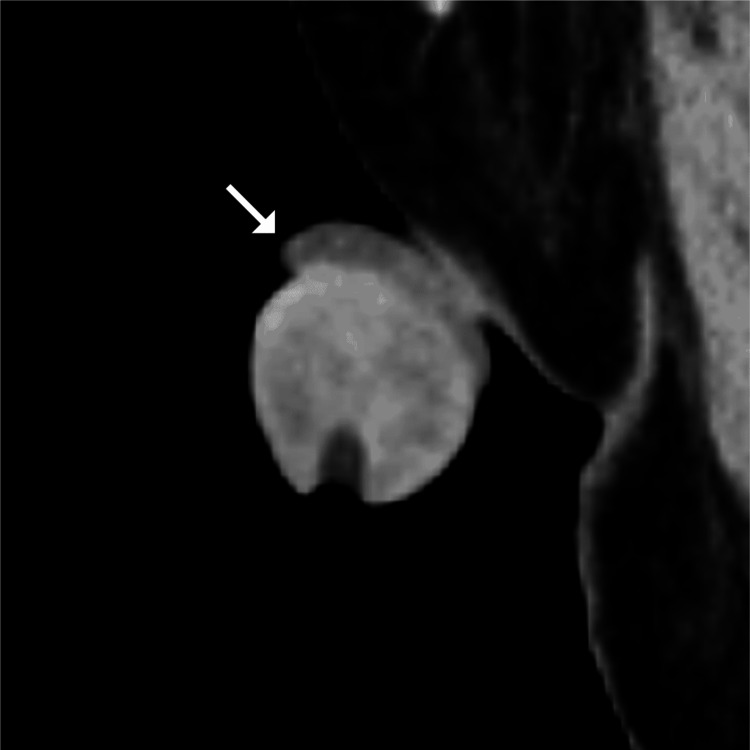
Sagittal contrast-enhanced CT of the penis. Sagittal contrast-enhanced CT of the penis shows a mildly thickened and hypoattenuating prepuce located proximal to the glans penis (long arrow).

**Figure 3:**
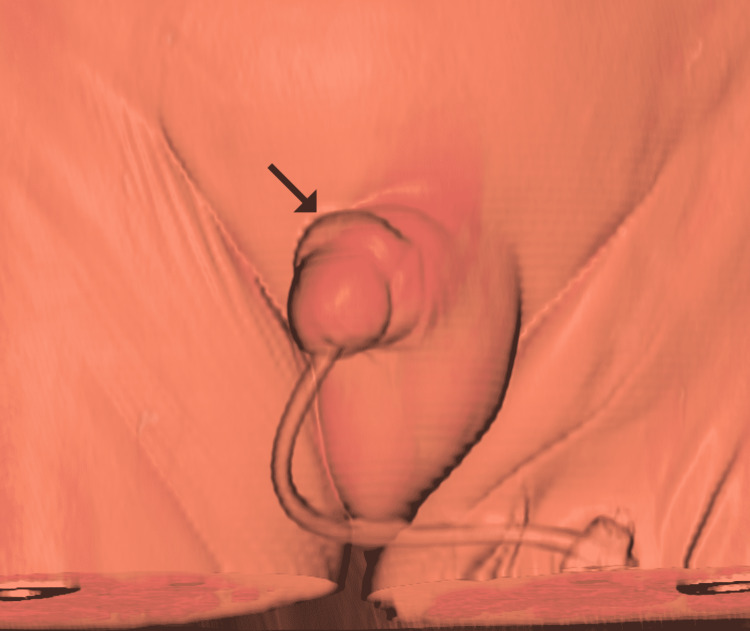
Three-dimensional volume rendering of the penis. A three-dimensional volume-rendered image better shows the preputial thickening and retraction (arrow), simulating the physical examination findings of paraphimosis.

**Figure 4:**
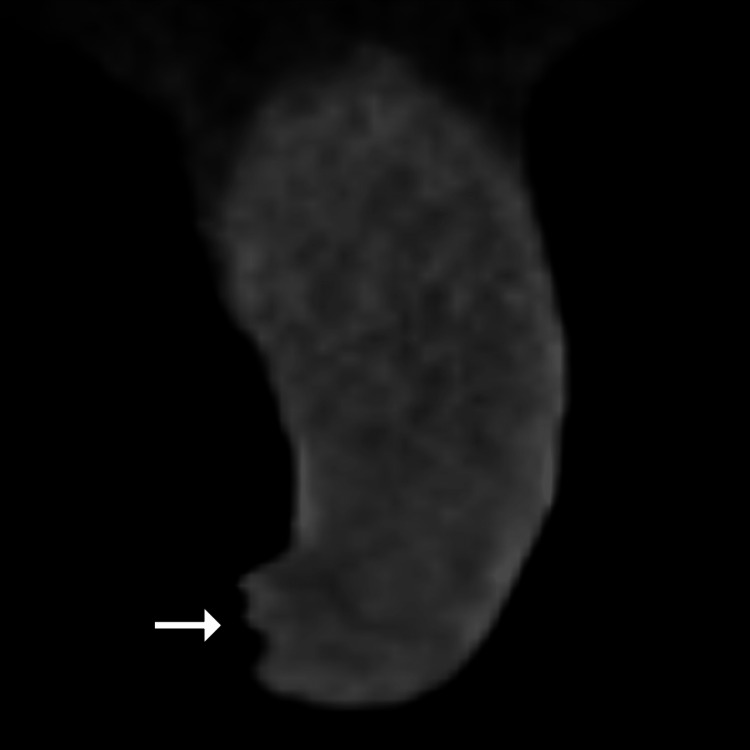
Coronal unenhanced CT in a different patient. An unenhanced coronal CT image in a different 84-year-old man without paraphimosis shows the normal relationship of the prepuce with respect to the glans penis (arrow).

Upon further discussion between urology and radiology, his CT was later reviewed with respect to his diagnosis of paraphimosis. Axial (Video 1), coronal (Figure 1), and sagittal (Figure 2) images demonstrated a relatively thickened prepuce which was located proximal to the corona of the glans penis and likely within the coronal sulcus. Compared to the surrounding skin of the penile shaft, the prepuce also appeared subjectively hypoattenuating, and was further assessed with a region of interest tool, yielding an average Hounsfield unit measurement of 13.1 for the prepuce compared to 24.4 in the adjacent subcutaneous tissues of the penile shaft. When scrolling through the multiplanar images, the prepuce appeared as a hypoattenuating, edematous halo or “collar” around the proximal glans penis. A three-dimensional volume rendering was also created (Figure 3) which simulates the physical examination findings of paraphimosis with penile edema and an entrapped prepuce. The CT examination of a different 84-year-old uncircumcised man without paraphimosis was provided for comparison (Figure 4) and shows the prepuce covering the glans penis in the expected fashion.

**Video 1:** Axial contrast-enhanced CT images of the pelvis and external genitalia. A video showing contrast-enhanced axial CT images of the external genitals from superior to inferior demonstrates the hypoattenuating prepuce located proximal to the glans penis as it appeared on the examination.

## Discussion

Paraphimosis is characterized by entrapment of the preputial skin behind the corona of the glans penis which eventually tightens around the penile tissues, leading to vascular compromise and causing edema and penile pain [1]. Incidence in adults is estimated at about 1% [3]. Undiagnosed paraphimosis can worsen in a continuous cycle of progressive vascular compromise, growing edema, and increasing difficulty in reducing the prepuce. If untreated it may eventually result in necrosis of the prepuce or erectile tissues and necessitate circumcision, or, rarely, penectomy [2]. When presenting in the medical setting, paraphimosis is often encountered when the prepuce has been retracted in an uncircumcised male patient to place a urethral catheter but has not subsequently been reduced after catheterization is complete [1,3]. In contrast, the closely named condition phimosis is a non-emergent entity which is characterized by the inability to retract the prepuce behind the glans penis [4]. Both conditions can affect males of all ages. It is possible that poor recognition of paraphimosis by clinical providers, lack of realization that it represents an emergency, or confusion with the similarly named but less problematic phimosis, may result in delays in care.

Ultrasound and MRI have been utilized successfully for multiple disorders affecting the male external genitalia, such as traumatic penile fractures, penile Mondor’s disease, and Peyronie’s disease [5]. Ultrasound is also the modality of choice for evaluating the testicles. However, imaging is not of prime importance when it comes to diagnosing and treating penile pathology for several reasons, and with respect to the rest of the body, imaging protocols and routine assessment are thus relatively scarce. CT, offering poor soft-tissue contrast, falls short in adequately assessing both male and female genitalia alike. MRI, while able to provide excellent image quality with an appropriate degree of soft tissue detail, is a costly and time-consuming examination when faster and cheaper (e.g., physical examination and ultrasound) options exist.

Paraphimosis is a condition that should be readily and easily diagnosed by physical examination. First-line conservative treatment measures involve an attempt at manual reduction of the prepuce [1]. As a result, diagnostic imaging is not performed for diagnosis and is not recognized as an adjunct examination by local or international guidelines [4], nor have a set of imaging findings been defined. The authors do not advocate for generating diagnostic imaging protocols to diagnose paraphimosis or incorporating them into routine practice. However, we suspect that if paraphimosis initially goes undiagnosed, radiologic studies performed for other reasons may potentially image the pathology and could contribute to expedited care when recognized by the radiologist. This may be of particular importance in poorly communicative, critically ill patients who may not be able to alert clinicians to the presence of penile pain and swelling. For this to happen, the imaging findings must be defined and radiologists must be aware of the condition and the possibility of its detection on imaging, as well as findings that may differentiate between other causes of penile edema, such as volume overload or infection.

In our case, we identified signs of edema as they present elsewhere in the body, mainly evidenced by relative hypoattenuation and thickening compared to adjacent normal tissues. Additionally, with the benefit of retrospective knowledge of the diagnosis, we were able to identify the abnormally retracted position of the prepuce with associated thickening and apparent hypoattenuation which we termed the “wet collar.” This presents a few challenges, however, as normal data have not been defined in this context. For example, preputial entrapment requires a dynamic assessment on the physical examination, and CT represents an image at a single point in time. The degree of preputial retraction which could be considered normal has not been elucidated. The level of relative hypoattenuation that lies within an acceptable range has not been described. Ultimately, the benefits of a comprehensive radiologic investigation into developing an optimal imaging protocol to assess paraphimosis must be weighed against the utility of the low-cost gold standard: the history and physical examination. However, this diagnosis may initially go overlooked in certain patient populations, and a radiologist’s knowledge of paraphimosis may allow its incidental detection for improved clinical outcomes.

## Conclusions

Paraphimosis is an emergent condition and imaging has no role in its routine evaluation. The authors do not advocate a change in practice at imaging centers or the development of imaging protocols for the detection of paraphimosis on imaging as an initial diagnostic test. However, it is a diagnosis that can initially go unrecognized, and patients will undoubtedly undergo radiologic studies for other reasons which may have a chance of imaging the pathology and allowing the radiologist to suggest the diagnosis. This can potentially lead to improved care and positively affect the patient’s condition. While the imaging findings we have presented and the “wet collar” sign we have proposed would require investigation to detail the breadth of normal imaging appearances of the prepuce and glans penis and to elucidate the performance profile of the “wet collar” sign as a marker for disease, we hope that with our report we have made the first steps toward bringing paraphimosis into the radiologist’s diagnostic awareness and given him or her factors to consider should he or she encounter it at the workstation.
